# Evaluation of a pre-set calcium sulphate dihydrate/hydroxyapatite bone graft extender in a rabbit posterolateral spinal fusion model

**DOI:** 10.1016/j.xnsj.2026.100955

**Published:** 2026-10-06

**Authors:** Gintarė Lukoševičiūtė, Filip Mrkonjic, Jintian Huang, Hadis Alidadi, Domantas Jakstas, Paulius Zasčiurinskas, Šarūnas Tarasevičius, Alfredas Smailys, Benny Dahl, Stefan Zwingenberger, Lars Lidgren, Magnus Tägil, Deepak Bushan Raina

**Affiliations:** a The Faculty of Medicine, Department of Clinical Sciences Lund, Orthopedics, Lund University, Lund, Sweden; b Department of Orthopedics and Traumatology, Lithuanian University of Health Sciences, Kaunas, Lithuania; c Department of Neurosurgery, Spinal Neurosurgery Unit, Lithuanian University of Health Sciences, Kaunas, Lithuania; d Faculty of Medicine, Lithuanian University of Health Sciences, Kaunas, Lithuania; e Department of Orthopedic Surgery, University of Copenhagen, Rigshospitalet, Copenhagen, Denmark; f Department of Orthopedics, Trauma Surgery and Rehabilitation Medicine, Greifswald University Hospital, Greifswald, Germany

**Keywords:** Biomaterials, Bone graft extender, Calcium sulphate, Hydroxyapatite, Spinal Fusion

## Abstract

**Background:**

Ceramic biomaterials are increasingly used as bone graft extenders (BGE) in spinal fusion surgery. The aim of this study was to evaluate the efficacy of a novel pre-set calcium sulphate/hydroxyapatite biomaterial (XERAFUSE) beads as a BGE using a well-established rabbit posterolateral lumbar spinal fusion model (PLF).

**Methods:**

Twenty-six skeletally mature rabbits underwent L4–L5 PLF using 2 treatment strategies: 1. Autograft alone (ABG) or 2. XERAFUSE co-implanted with ABG in 1:1 volume ratio (XER + ABG). Evaluation of spinal fusion was performed post-mortem at 4-, 8-, and 12-weeks for XER + ABG group and at 12-weeks for the ABG group. Evaluation of spinal fusion was performed using radiography, micro–computed tomography (micro-CT), manual palpation and histological analysis.

**Results:**

Based on the micro-CT based histomorphometric analysis, the fusion mass in XER + ABG group demonstrated an increase in bone volume (59%–72%) and reduction in material volume (41% vs 28%) between the 4- and the 12-week time points. The bone volume in the XER + ABG group (850 ± 68.4 mm³) was significantly higher than in the autograft comparator group (468.9 ± 105.3 mm³) at 12-weeks. The outcome from qualitative analysis at 12-weeks using planar radiography, 2D and 3D micro-CT imaging, manual palpation and histology demonstrated 86% healing in XER + ABG group while 57% specimens demonstrated healing in the ABG group.

**Conclusions:**

The results from our study indicate that XERAFUSE is a safe and efficient BGE, demonstrating significantly greater bone volume than autograft control, and fusion rates consistent with the model. The use of XERAFUSE as a BGE may reduce dependence on large volumes of autologous bone graft in spinal fusion surgery while maintaining or improving fusion performance. These findings support the translational potential of calcium sulphate/hydroxyapatite composite biomaterials as clinically relevant BGE in spinal arthrodesis procedures.

## Introduction

Calcium phosphate (CaP) – based biomaterials have been clinically used for filling of bone defects since the early 20th century due to their chemical similarity to hydroxyapatite in bone [1]. Although autologous bone graft remains the gold standard, synthetic resorbable bone grafts are a compelling alternative when the amount and quality of local or iliac crest autograft is limited, and harvesting is hampered with complications [2].

In the last decades, ceramic biomaterials have evolved from prefabricated blocks to injectable, in situ setting composite systems, ion-substituted formulations and biologically functionalized constructs [[3], [4], [5]]. β-tricalcium phosphate (β-TCP), hydroxyapatite (HA), calcium sulphate (CaS) and their combinations have been reported with various outcome in preclinical fusion studies and have only recently been approved as combinations with 50% bone graft as extenders (BGE) in spinal fusion applications [[6], [7], [8]]. The synthetic bone grafts provide structural support and act as a scaffold but with limited biological signalling and need to be combined with an autograft [9]. This limitation is particularly critical in posterolateral lumbar fusion (PLF) where bone must form in a partially extraosseous environment with significant muscle-material crosstalk [10].

The rabbit PLF model is a well-established spinal fusion model recognized by regulatory authorities, including the U.S. FDA for evaluation of biomaterials and biological therapies intended for use in spinal fusion surgery. The evaluation of fusion mass in this model depends on the assessment modality used, as spinal fusion outcomes are best interpreted using a multimodal approach, given that no single technique fully captures the fusion process. Fusion rates reported at 12-weeks in rabbit posterolateral fusion models using commercially available bone graft substitutes, as assessed by manual palpation, radiography, and micro–computed tomography are shown in Table 1.

**Table 1:** Fusion rates reported in rabbit PLF model at 12-weeks using commercially available bone graft substitutes

Study	Name	Primary composition	Manual assessment	X-ray	Micro-CT
Walsh et al., 2009[11]	FormaGraft® Collagen Bone Graft Matrix	Biphasic calcium phosphate (HA + β-TCP) + Bovine Collagen Type I	83.3%	-	-
van Dijk, 2019 [12]	MagnetOs® Granules	Biphasic calcium phosphate (HA + β-TCP) with submicron surface characteristics + Synthetic polymer carrier	75%	87.5%	100%
Conway et al., 2021 [13]	Catalyst™ Putty	Calcium phosphate (nSiCaP) with bioactive surface characteristics + Synthetic polymer carrier	87.5%	62.5%	62.5%
Walsh et al., 2025 [14]	ATTRAX® Scaffold	Biphasic calcium phosphate (HA + > 90% β-TCP) + Bovine Collagen Type I	75%	75%	75%
Smucker et al., 2012† [15]	MASTERGRAFT™ Strip	Biphasic calcium phosphate (HA + β-TCP) + BMA	75%	-	-

† Fusion rate reported at 8 weeks.

There are significant clinical challenges in achieving solid bony posterolateral spinal fusion in humans using autografts, and nonunion rates of 10–40% have been reported, although mentioned success rates are up to 90% [16,17]. Clinical studies with noninvasive radiographic and CT-based follow-up studies have failed to clearly differentiate between new ingrowing bone and the implanted ceramic graft extender [18]. This is why a rabbit model of non-instrumented posterolateral spinal fusion described by Boden and coworkers has been acknowledged by FDA, representing a distinct biological environment mirroring a worst-case clinical fusion scenario and not be confused with other large-animal models reporting much higher fusion rates [19]. Contrary to the clinical scenario where a balance of radiation dose and image quality is of significance for patient safety, high-resolution micro-CT imaging can be applied in preclinical studies. Micro-CT has been used to differentiate between bone and bone mineral-containing material allowing histomorphometric measurements in a 3D volume using available software [20].

XERAFUSE is a novel pre-set ready to use bone mineral granule containing 60% calcium sulphate dihydrate (CSD) embedding 40% particulate hydroxyapatite (HA). Calcium sulphate–based materials have been reported to resorb within 4–8 weeks, with the calcium released during dissolution proposed to promote local HA precipitation and stem cell recruitment [21,22]. The CSD local HA particles serve as a framework for new ingrowing bone filling the porous structure [23,24]. We hypothesize that a precision-engineered CSD/HA scaffold with a unique mechanism of action, with early calcium-driven stem cell recruitment, and HA retention will lead to guided bone regeneration and improve overall bone quality in the fusion mass in PLF.

This study aims to evaluate:

1. A biphasic CaS/HA based bone mineral granule as BGE in a preclinical rabbit posterolateral fusion model in comparison with autologous bone. Evaluation is carried out using radiography, manual palpation, and histology individually using each technique as well as combined global outcome.
2. Could high-resolution micro-CT imaging separate the BGE from native bone and thereby evaluate the dynamics of material resorption and bone remodelling in the fusion mass.

## Materials and methods

### Experimental materials

Autograft (ABG) bone harvested from the iliac crest was used as the positive control group. Autograft was coimplanted with the material described below in a 1:1 volume ratio (50/50). XERAFUSE granules (cylinder shaped, approximately 2 mm diameter and 2–4 mm length) were used as a bone graft extender, implanted with an equal volume of autologous bone as sequential layers. XERAFUSE comprised of 60% w/w of CSD embedding 40% w/w of HA and is referred to as XER hereafter.

### Scanning electron microscopy (SEM)

The XER material was examined by scanning electron microscopy (JEOL JSM-7800F, Tokyo, Japan). Granules were fractured manually between the index finger and thumb, mounted on aluminium stubs using conductive carbon adhesive tape, and sputter-coated with a 5–10 nm palladium/gold (Pd/Au) layer prior to imaging. SEM was used to assess surface morphology and pore architecture qualitatively; porosity, pore size and interconnectivity were not quantified.

### Experimental design and animals

Twenty-six adult female New Zealand White rabbits, each weighing approximately 2.8 to 3.2 kg, were obtained from Charles River Laboratories (France) and included in this study. Following a 7-week acclimatization period at the animal facility, the animals had reached an age of >7 months. Skeletal maturity was confirmed by radiographic documentation of proximal tibial growth plate closure prior to surgery. Single-level posterolateral lumbar fusion was performed at L4–L5 level, bilaterally, following the original surgical protocol described by Boden et al. [19]. For each time point of 4-, 8- and 12-weeks n=6, 6 and 7 rabbits were used, respectively (Table 2). The sample size was based on the results and standard deviations of previous studies using this model. A sample size of n=7 was used for the ABG group and evaluation was performed only at the terminal time point of 12 weeks. All animal procedures were carried out according to the institutional guidelines at the Lithuanian University of Health Sciences, Kaunas, Lithuania and were approved by the animal ethics committee in Lithuania (approval number: G2-277).

**Table 2:** Distribution of experimental animals across study endpoints

Test group	4 weeks	8 weeks	12 weeks
CSD/HA + ABG	5	6	7
ABG (positive control)	-	-	7

### Surgical technique

The surgical approach was in accordance with Boden et al. as well as ASTM F3207-17 guidelines [19,25]. Briefly, iliac crest was exposed and corticocancellous bone graft was harvested and morselized to 1–4 mm chips using ronguers. Total volume of autograft alone or autograft combined with BGE was 3 mL, a standard amount stated to be needed for fusion [25]. For the ABG group, 3 mL of autologous bone was used on each side. In the case of XER + ABG group, 1.5 mL XER granules and 1.5 mL autograft (3.0 mL/ side) were measured separately and subsequently coimplanted using a 3 mL syringe.

All the surgeries were carried out in aseptic conditions. The rabbits were anesthetized by using 100 mg/mL of ketamine at a volume of 0.3 mL and then maintained with isoflurane 2%. Rabbits were placed prone on the operating table and surgically prepped with 70% povidone-iodine solution. After skin infiltration with 2% lidocaine, a midline skin incision of approximately 6 cm was made from L1 to the sacrum. The underlying fascia was then incised separately followed by blunt dissection of multifidus and longissimus muscles to access the L4 and L5 transverse processes. An exposed central region of the transverse process was decorticated using high-speed burr, ensuring a gap of approximately 1 cm from the vertebral body. In XER + ABG group a total of 1.5 mL of autograft was placed in the paraspinal bed between the decorticated levels using a 3 mL open-end syringe, followed with 1.5 mL of XER on top. In autograft only group, 3 mL of harvested iliac crest graft was placed between L4 and L5 transverse processes. The muscle planes and fascia were closed using 3-0 Vicryl, and then the skin was stapled with surgical staples. Dorsoventral radiographs were taken postoperatively to confirm appropriate material placement.

Postoperative care was conducted according to institutional animal welfare protocols. All animals received perioperative antimicrobial prophylaxis with one dose of enrofloxacin (10 mg/kg) administered intramuscularly. Postoperative analgesia was maintained with ketoprophen 100 mg/mL at a volume of 0.06 mL for at least 72 hours postop. Throughout the study, animals were individually caged, had access to rabbit food and water and were monitored daily for signs of pain and discomfort. Operated sites were observed daily for the entire period to ensure proper healing of the implant sites and for any clinical signs of irritation/toxicity.

The rabbits were humanely killed at different times postoperatively (4-, 8- and 12-weeks). The lumbar spines were removed en bloc, containing L1–S1 segments and denuded of soft tissue. Prior the general integrity of the incision was noted, along with any evidence of macroscopic reaction of the underlying subcutaneous tissues. Fusion mass was evaluated by dorsoventral radiographic analysis, micro-CT imaging, and manual palpation. Histological analysis was performed to assess the tissue response to the graft material.

### Postoperative radiography

Following necropsy, dorsoventral lumbar digital radiographs were taken using a GXR-SD Series DR system (DRGEM, South Korea). Digital images were processed using the manufacturer’s integrated software and the DICOM data was converted to JPEG images using IMAIOS medical viewer. Scoring was performed in a blinded manner by 3 independent observers based on the presence of continuous radiopaque bridging indicating fusion and radiolucent features indicating nonfusion. The grade of fusion for each sample was described referencing the Lenke 4-point grading scale, as grade A to D, Table 3.

**Table 3:** Radiographic fusion grading system as described by Lenke et al. [26]. Fusion was categorized as: (A) definitely solid; (B) possibly solid; (C) probably not solid; and (D) definitely not solid, based on radiographic appearance

Grade	Description
A	Large, continuous bilateral fusion masses
B	Large unilateral fusion mass with a smaller or incomplete contralateral fusion mass
C	Small, thin bilateral fusion masses
D	Bilateral graft resorption or fusion masses with radiographic evidence of pseudarthrosis

### Manual palpation

Mechanical stability of the treated segment was assessed comparing movements to the adjacent cranial and caudal levels. The L4–L5 motion segments were further graded by 3 independent blinded observers as “fused,” if no detectable motion at the segment was detected in flexion and extension. The fusion was graded as “not solid,” if motion was present [25].

### Micro computed tomography

High resolution images were obtained using a micro-CT scanner (MI Labs, Netherlands) at 65 kV, 0.13 mA, 75 ms exposure time and a 0.4 mm aluminum filter. Micro-CT scans were reconstructed using PMOD software (version 4.005; PMOD Technologies Ltd., Zurich, Switzerland) with an isotropic voxel size of 30 µm. Quantitative morphometric analysis was performed and 3-dimensional visualizations created using Dragonfly software (Dragonfly 3D World, version 1.14.4.1) following a workflow developed by the authors, described below. The images were also evaluated in the axial, coronal and sagittal planes and determined as fused or not in a blinded manner to treatment and time by 3 independent observers. Micro-CT fusion scoring applied the same criterion as plain radiographs, with successful fusion defined as continuous radiodense bridging bone consistent with Grade A of the Lenke grading system, Table 3.

### Dragonfly analysis

Quantitative morphometry of the ex vivo micro-CT images of rabbit spines was done using Comet Technologies Canada Inc. Dragonfly 3D World. Version 1.14.4.1 software. Segmentation was performed using global intensity thresholding, separating mineralized tissue (bone and residual biomaterial) from soft tissue and background. A threshold of 1900–6500 HU, selected based on histogram analysis and visual assessment and applied consistently across all samples and experimental groups. The fusion area between the L4–L5 transverse processes was selected bilaterally and segmented as bone tissue (residual autograft and newly formed bone) or biomaterial using the Dragonfly Segmentation Wizard, a trained classifier that assigns voxels based on thresholding and morphological characteristics. The analysis was performed independently by 2 evaluators: each manually labelled representative slices spanning the volume of interest into bone, biomaterial and background classes, and reviewed the resulting classification plane by plane, Fig. 1 (B). Quantitative measurements were obtained by and compared together, with cumulative variance of < 15%. A standardized set of parameters, including bone volume (mm³ and %) and material volume (mm³ and %), was obtained for final analysis. Three-dimensional pseudoholotomographic reconstructions of the fusion region were generated for each specimen using the “Bone” preset, with distinct color coding applied to differentiate bone and biomaterial across multiple viewing axes, Fig. 1 (A).

**Fig. 1:**
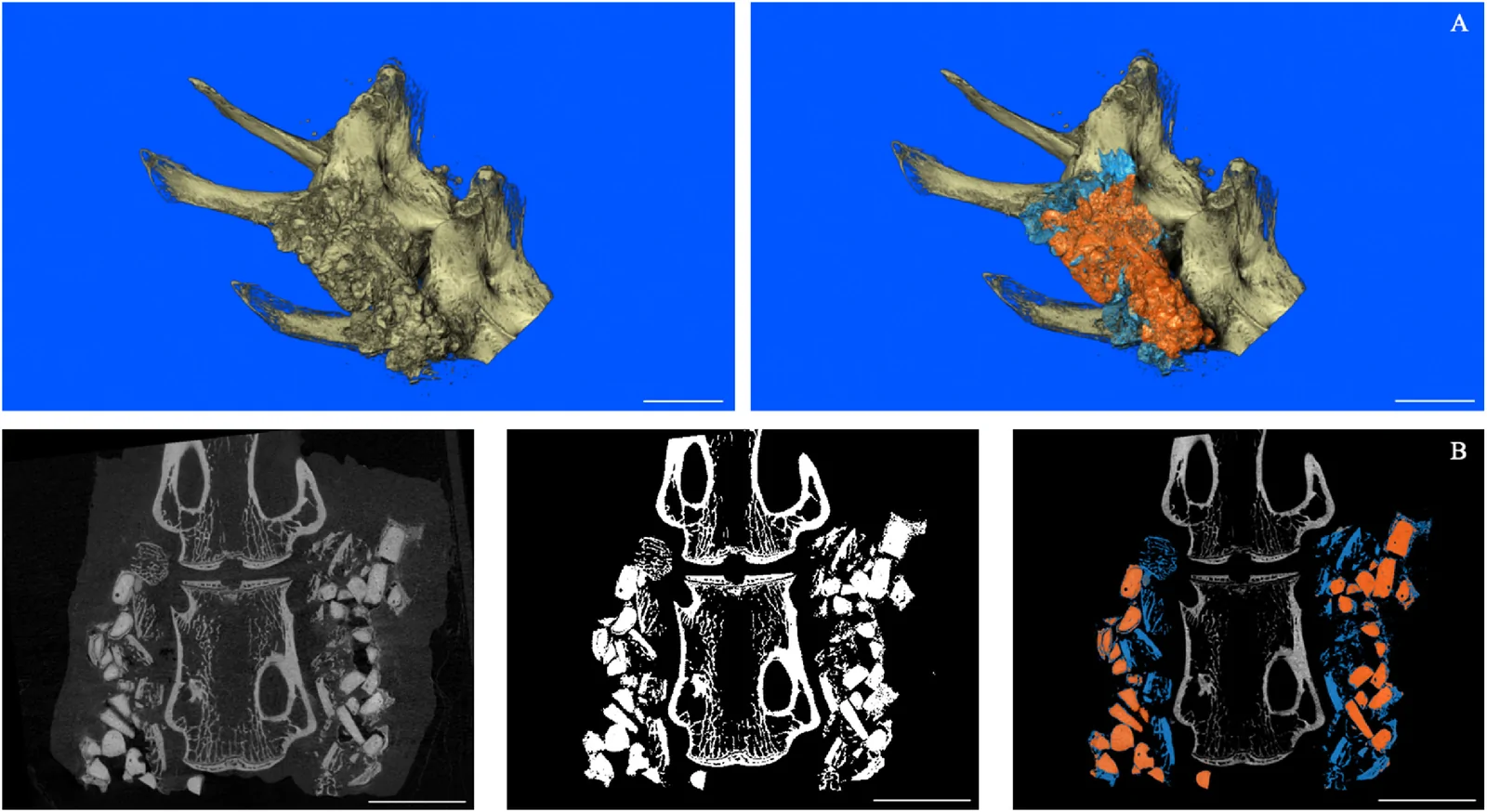
Dragonfly-based analysis of rabbit spine micro-CT images. (A) Three-dimensional pseudo-holotomographic reconstruction of the L4–L5 fusion region generated using the “Bone” preset, with color coding distinguishing bone tissue (blue) and biomaterial (orange). (B) Coronal micro-CT images of the spine shown before thresholding, after global intensity thresholding, and after segmentation into bone tissue (blue) and biomaterial (orange). Scale bars, 1 cm.

### Histological evaluation

All specimens were fixed in neutral buffered formalin (10%) for a minimum of 48 hours and then decalcified in 10% formic acid solution until decalcification was deemed complete followed by thorough rinsing with water up on completion. The decalcified and dehydrated samples were embedded in paraffin, and vertebrae were sectioned in the sagittal plane using a Thermo Scientific HM 355S microtome (Thermo Fisher Scientific, Waltham, MA, USA) to obtain 7-µm-thick sections. At least 3 sections of the fusion area within a thickness of 300 µm were stained (haematoxylin and eosin [H&E]) and examined using an Olympus light microscope (Olympus, Japan). Slides were digitized using an Olympus VS200 slide scanner (Olympus Corporation, Tokyo, Japan) and analyzed using QuPath (v0.6.0) software for fusion and tissue characteristics.

### Statistical analysis

Sample size was empirically calculated based on previous studies using the rabbit PLF model with similar study aims. Statistical analysis was performed using GraphPad Prism (v5, GraphPad Software Inc.). Normality of the data distribution was assessed prior to analysis. Grading of spinal fusion (manual palpation, radiographic fusion, and micro-CT fusion) were compared between groups using Fisher’s exact test. Student t-test was used to compare Micro-CT fusion mass data for appropriate groups. Statistically significant differences were considered for values of p<.05.

## Results

### Material characterization

The surface morphology and pore architecture of XER granules were examined using scanning electron microscopy. The material demonstrated a roughened surface composed of interconnected micron-sized particles interspersed with micro-sized pores, Fig. 2 (A-B). At 5000 × magnification, needle-like CSD crystals and micron-sized angular HA grains were clearly distinguishable, Fig. 2 (C), indicating that this microporosity arises from the packing of the 2 crystalline phases during setting and is therefore present in the granules as manufactured.

**Fig. 2:**
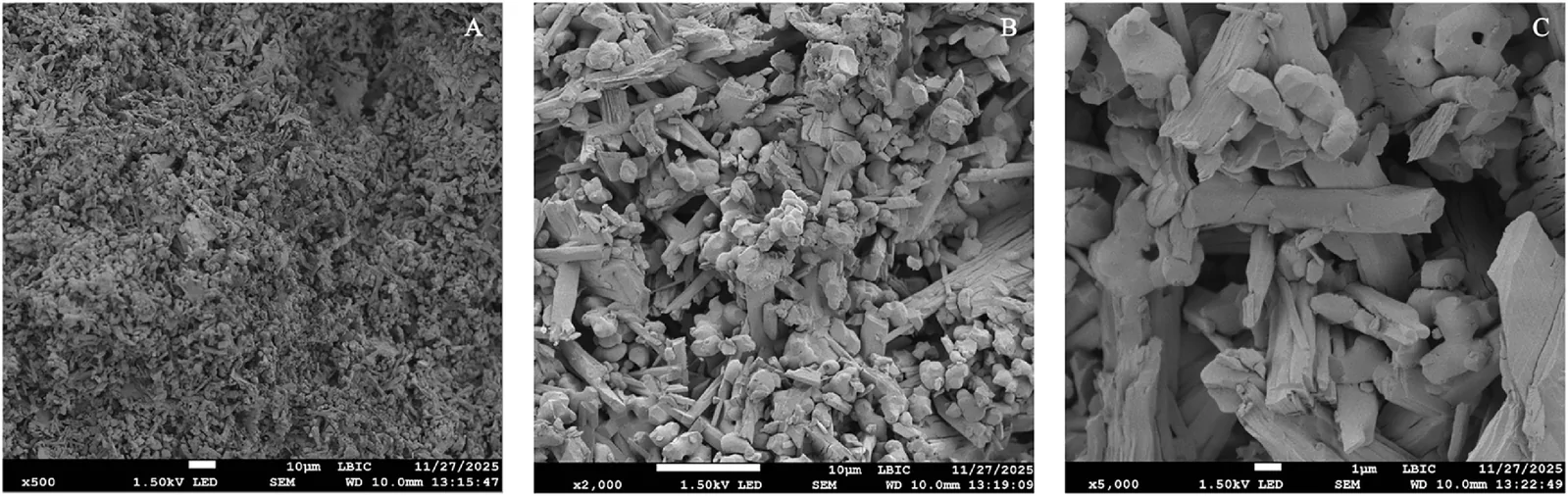
SEM images of pre-set CSD/HA granules demonstrating structure and surface topography at low (A, x 500), moderate (B, x 2000) and high (C, x 5000) magnification.

### Surgery

All rabbits tolerated the surgical procedure well. Animals revived from surgery within 4 to 6 hours, and fully recovered within 2 weeks with no complications, such as hind leg weakness, or obvious surgical site infection. At the time of tissue harvest, no adverse organ reactions were identified. One animal died 5 days postoperatively, with no obvious signs of local surgical site inflammation or gross abnormalities observed during necropsy except excessive hemorrhaging around the iliac crest.

### Manual palpation

Fusion of the spines was assessed by manual palpation with fusion rate (%) generally increasing with increased implantation time for each group, Table 4. At 4, 8, and 12 weeks, manual palpation demonstrated fusion rates of 40%, 67%, and 86% in the XER + ABG group, respectively. At 12 weeks XER + ABG was also compared to ABG group, with ABG showcasing fusion rate of 71.4%. No statistically significant differences were observed between groups at any timepoint.

**Table 4:** Proportion of animals classified as fused based on manual palpation, radiography, micro-CT and histology at different time points

Test group	Manual palpation	X-ray	Micro-CT	Histology	Overall fusion†
CSD/HA + ABG, 4-weeks	40% (2/5)	80% (4/5)	60% (3/5)	60% (3/5)	40% (2/5)
CSD/HA + ABG, 8-weeks	67% (4/6)	83% (5/6)	83% (5/6)	50% (3/6)	33% (2/6)
CSD/HA + ABG, 12-weeks	86% (6/7)	100% (7/7)	100% (7/7)	100% (7/7)	86% (6/7)
ABG, 12-weeks	71% (5/7)	57% (4/7)	71% (5/7)	71% (5/7)	57% (4/7)

† Fusion rate of samples classified as fused by all evaluation methods.

### Radiography

Representative radiographs for the 4 study groups at the endpoints of 4, 8 and 12 weeks are shown in Fig. 3. Radiographic evidence of fusion was seen already by 4 weeks, Fig. 3 (A). Distinct edges of biomaterial started to blur and were not as clearly seen in any of the implanted grafts at 8- Fig. 3 (B) or 12-weeks Fig. 3 (C). Radiographs show progression towards a solid fusion with 80%, 83%, and 100% evaluated as bilaterally fused at 4, 8, and 12 weeks in XER + ABG group, respectively. In contrast, radiographic fusion rate was 57% in the autograft control group at 12 weeks, Table 4, Fig. 3 (D). Overall, the mineral particles were coarsely arranged, and the implant had a strong radiopacity, which ultimately made it difficult to critically assess new bone within the graft materials based on radiographs alone. A full panel of radiographs for all endpoints are provided in Supplementary Information, Fig. S1.

**Fig. 3:**
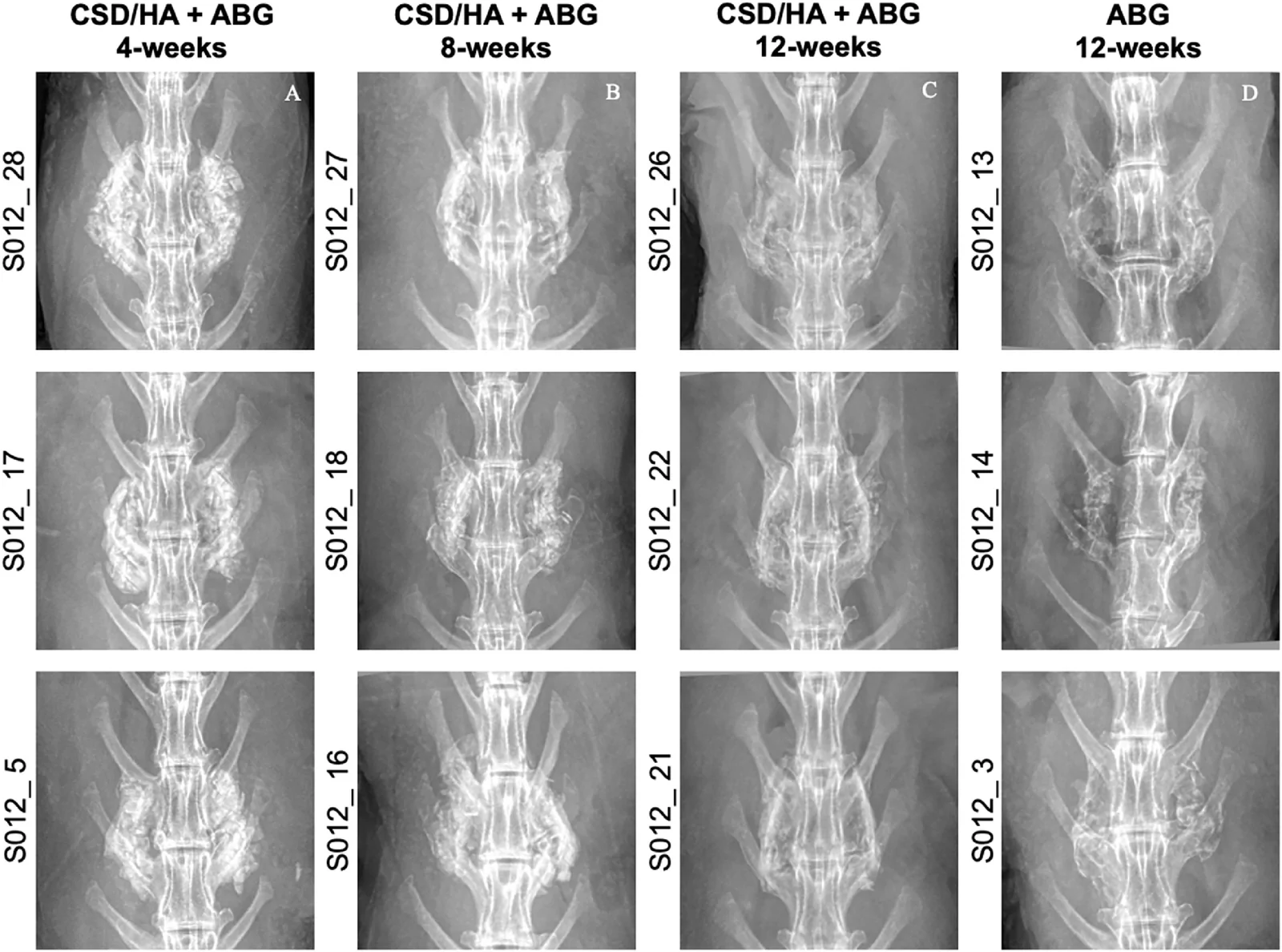
Representative radiographs demonstrating fusion progression in the CSD/HA + ABG group (A–C) and comparison with autologous bone graft at 12 weeks (D). (A) CSD/HA + ABG group at 4 weeks, showing early radiographic evidence of fusion; (B) CSD/HA + ABG group at 8 weeks, with reduced visibility of biomaterial margins; (C) CSD/HA + ABG group at 12 weeks, demonstrating further consolidation toward solid fusion.

### Micro-computed tomography

Fusion between the L4–L5 transverse processes was also evaluated using 3-dimensional micro-CT reconstructions and assessed in 3 orthogonal planes with the Dragonfly application (Dragonfly 3D World, version 1.14.4.1), Fig. 4. Representative 3D micro-CT pseudoholotomographic reconstructions and their changes at 4-, 8-, and 12- weeks for the XER + ABG group and autograft control group, are shown in Fig. 5. The fusion rate was 60% at 4 weeks, 83% at 8 weeks, and 100% at 12 weeks in the XER + ABG group, compared with 71% at 12 weeks in the autograft group, as evaluated by micro-CT, Table 4.

**Fig. 4:**
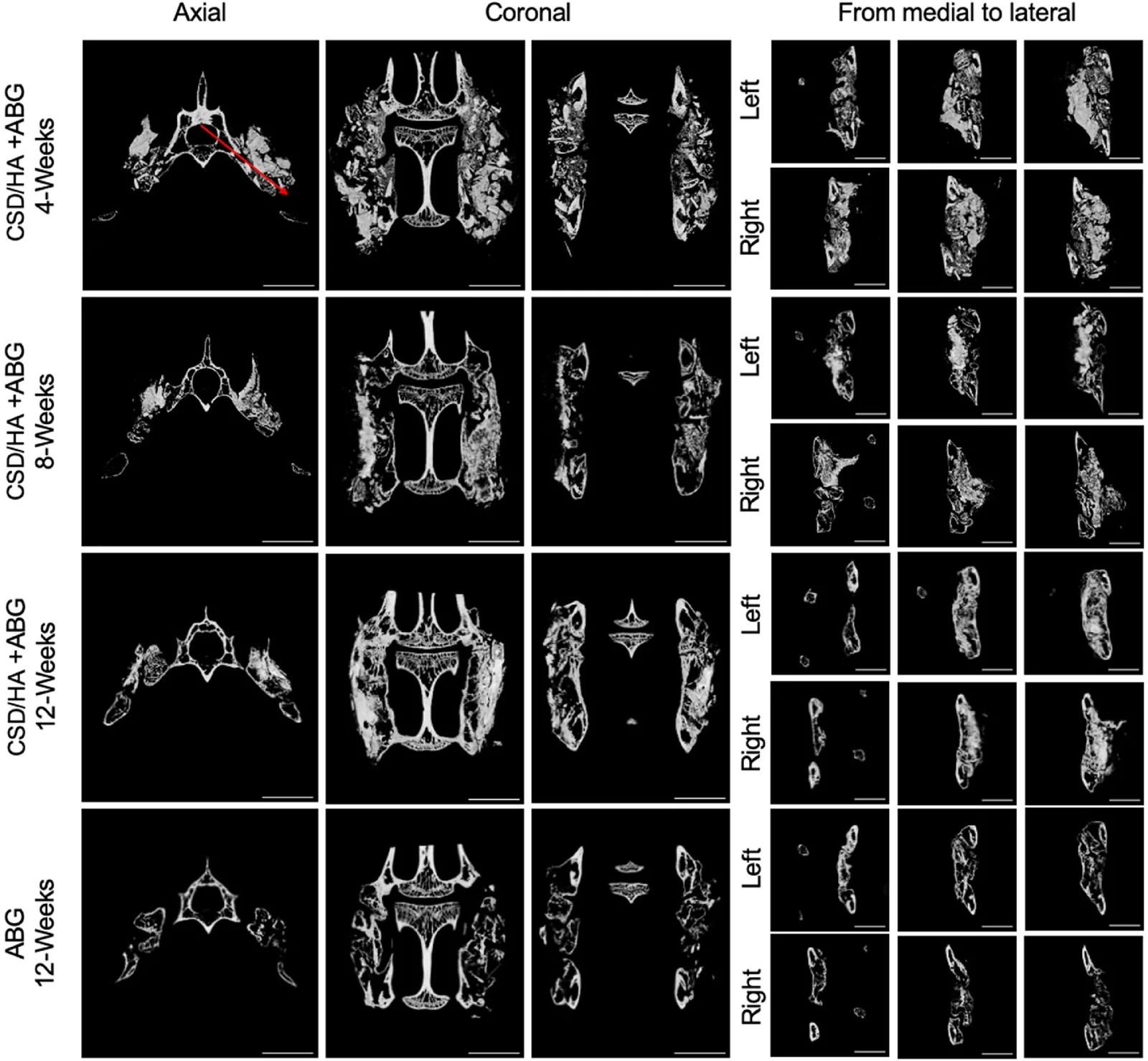
Micro–computed tomography multiplanar reconstruction. The axial view is provided for spatial reference, with the red arrow indicating the direction of sectioning. The coronal plane is represented by the first and last images of the evaluation sequence. The sagittal plane includes 3 images obtained at 30-slice intervals, demonstrating fusion progression from medial to lateral side. Scale bars, 1 cm.

**Fig. 5:**
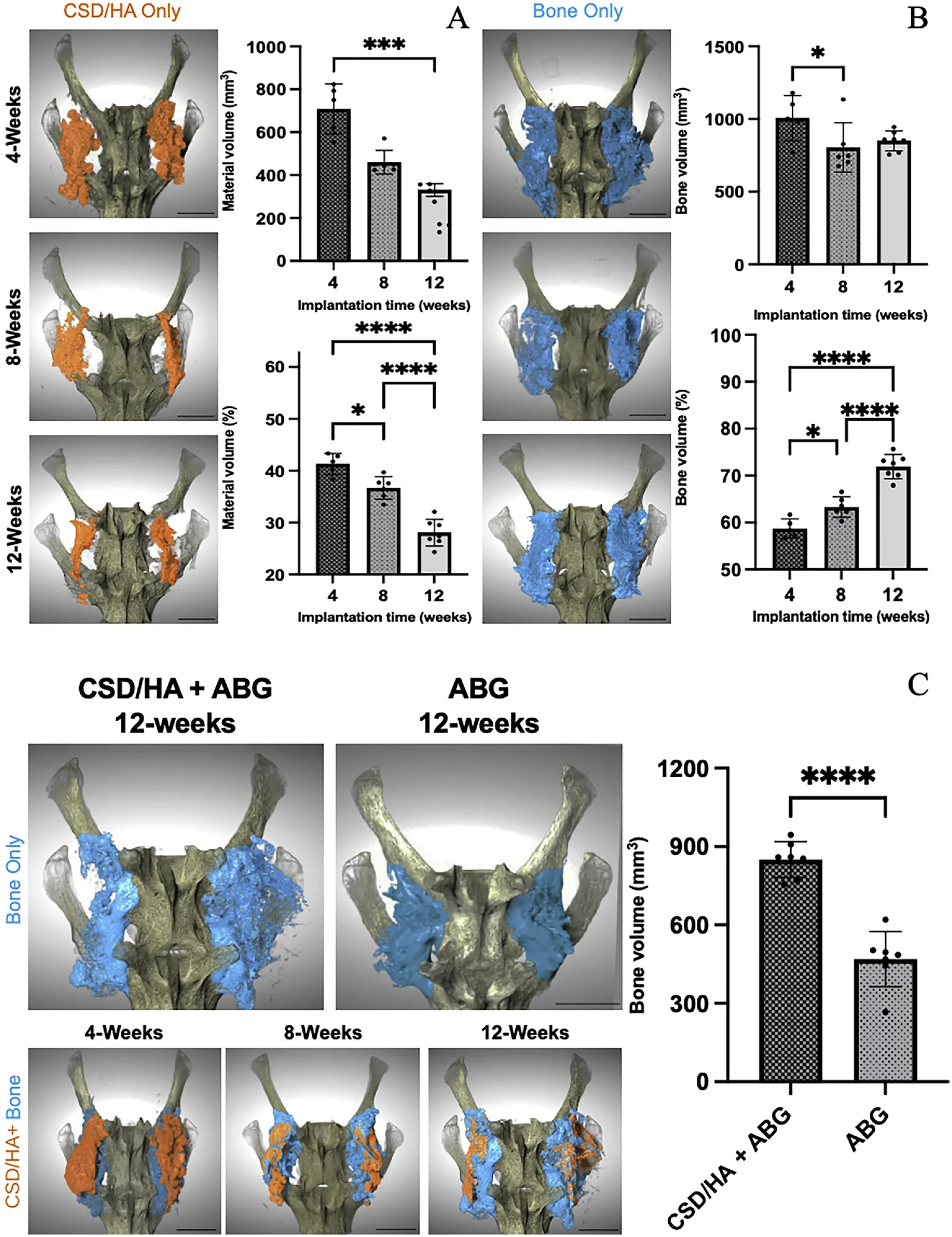
Micro-CT–based evaluation of fusion mass evolution. (A, B) Representative 3-dimensional pseudo-holotomographic reconstructions with corresponding quantitative analysis illustrating changes in biomaterial (A) and bone (B) within the CSD/HA + ABG group at 4, 8, and 12 weeks. (C) Comparison of bone volume between the CSD/HA + ABG and ABG groups at 12 weeks and changes in fusion mass composition within the CSD/HA + ABG group over time, with representative 3-dimensional pseudo-holotomographic reconstructions shown for reference. *p<.05; ***p<.001; ****p<.0001; Scale bars = 1 cm.

Fusion mass was quantitatively separated into remaining material volume and bone volume (residual autograft and newly formed bone) as shown in Fig. 1. Biomaterial volume (mm³) decreased significantly over the 12-week period, from overall of 749.8 mm³ (IQR: 586.9–808.5) at 4 weeks to 342.3 mm³ (IQR: 309.1–356.7) at 12 weeks (p=.001), Fig. 5 (A). Overall, at 4 weeks, biomaterial made 41.3±2.0% of the fusion mass and progressively decreased in time, with 36.7±2.2% fraction at 8 weeks and 28.1±2.6% at 12 weeks, Fig. 5(A).

Total bone volume (mm³) decreased significantly from 4 to 8 weeks (median 1001.7 mm³, IQR: 874.9–1140.0 vs. 742.1 mm³, IQR: 696.3–904.5; p=.041), before increasing again at 12 weeks (median 859.2 mm³, IQR: 790.9–907.4), Fig. 5(B). However, in the fusion mass, the fraction of bone in relation to biomaterial increased stably, with 58.7±2.0% bone at 4 weeks, 63.3±2.2% at 8 weeks and 71.9±2.6% at 12 weeks, Fig. 5 (B).

At the 12-week endpoint, overall bone volume was compared between the XER + ABG and ABG groups and was significantly greater in the XER + ABG group (850.0±68.4 mm³ vs. 468.9±105.3 mm³, p<.0001), Fig. 5 (C). Compositionally, at 4 weeks, micro-CT demonstrated XER granules predominantly located on the surface of the autograft, whereas at 8 and 12 weeks the granules appeared increasingly integrated within a trabecular network, with progressive loss of spherical morphology, Fig. 5 (C). A full panel of micro-CT reconstructions for all endpoints are provided in Supplementary Information, Fig. S2.

### Histology

Low-magnification decalcified histological images of the L4–L5 fusion site stained with H&E provide an overview of the healing response in the XER + ABG group at 4, 8, and 12 weeks, Fig. 6 (A1–C1). By 12 weeks, continuous trabecular bone with bone marrow spaces bridging the transverse processes was observed in 100% (7/7) of samples in the XER + ABG group, compared with 71% (5/7) in the ABG group, Fig. S3, Table 4.

**Fig. 6:**
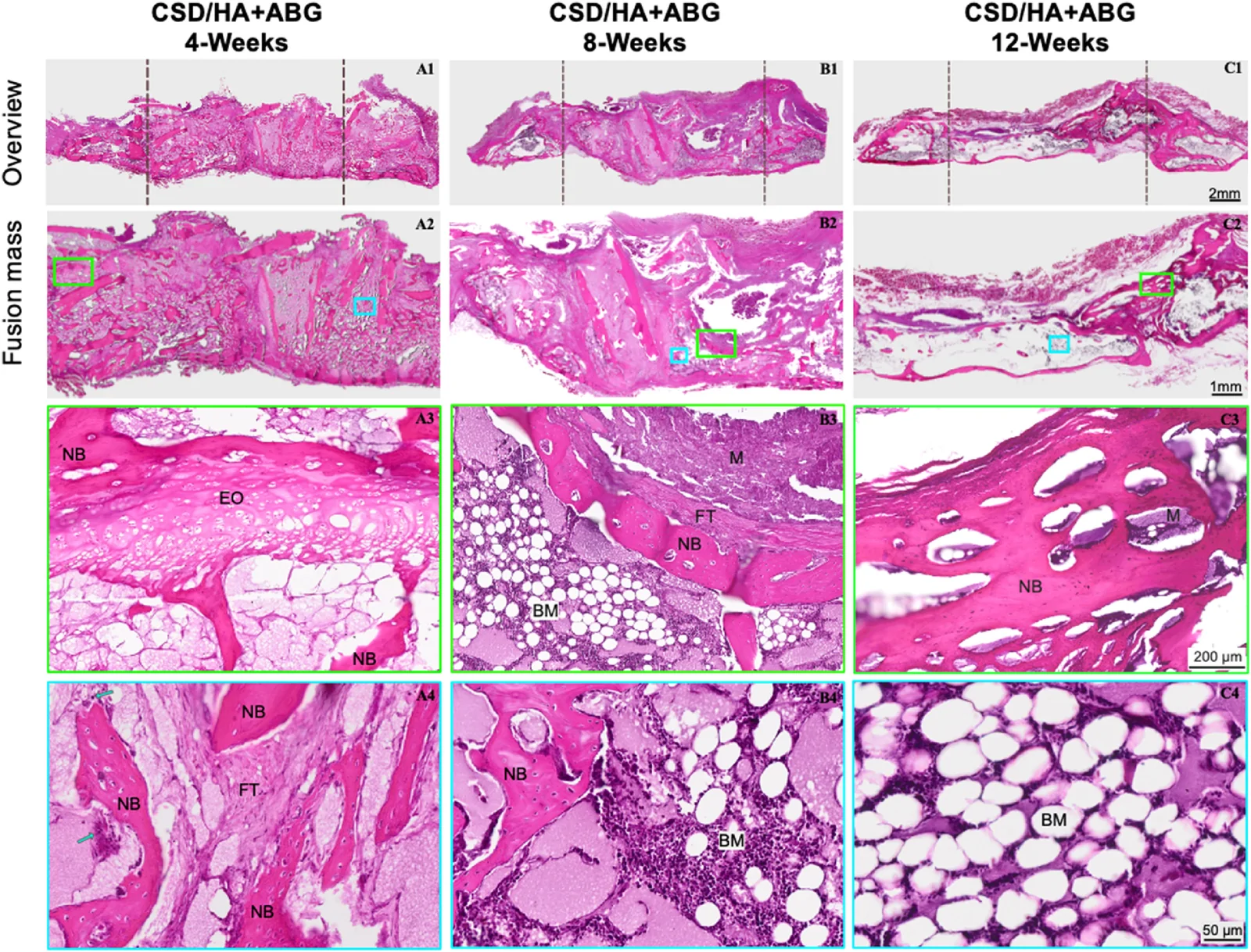
Representative sagittal histological images of the posterolateral fusion site between the L4–L5 transverse processes at 4 (A), 8 (B), and 12 (C) weeks after surgery in the CSD/HA + ABG group, stained with H&E. NB: new bone, EO: endochondral ossification, FT: fibrous tissue, M: material, BM: bone marrow. Green and light blue rectangles indicate regions selected within the fusion mass and shown at higher magnification (200 µm and 50 µm, respectively). (A3) Early new bone formation associated with endochondral ossification. (A4) New bone with interspersed fibrous tissue and mild inflammatory cell infiltrate (light blue arrows). (B3) Residual biomaterial with fibrous tissue and new bone ingrowth. (B4) Trabecular bone with emerging bone marrow spaces. (C3) Continuous trabecular bone bridging the fusion mass with minimal residual biomaterial. (C4) Bone marrow present centrally.

At 4 weeks in XER + ABG, autologous bone graft and biomaterial maintained space in the defect area, so the center of the defect did not collapse Fig. 6 (A2). New bone formation was observed adjacent to the transverse processes and within the implanted material, with chondroblastic tissue regions indicating endochondral ossification pathway Fig. 6(A3). A mild inflammatory response was observed adjacent to autograft particles and biomaterial, Fig. 6 (A4) which diminished at 8- and 12- weeks, supporting the biocompatibility of XER.

In the 8-week group, the previously biomaterial and autograft filled fusion mass showed progression with areas new bone formation developing adjacent and between the biomaterial granules, Fig. 6 (B3). A trabecular bone network with developing bone marrow spaces was present and variably extended across the intertransverse process space, Fig. 6 (B4).

This progression in healing extended to the 12-week time point, with a denser trabecular bone network and increased bone marrow spaces observed, Fig. 6 (C3–4).

Overall fusion rates increased over time, with 60% (3/5) of samples evaluated as fused at 4 weeks, 50% (3/6) at 8 weeks, and 100% (7/7) at 12 weeks (Fig. S3), Fig. S3, Table 4.

At 12 weeks, histological findings in the XER + ABG group were comparable to those in the ABG group, with new bone formation and bone marrow spaces observed within the fusion mass. Despite overall similarities, distinct group-specific features were observed: residual biomaterial remained in the XER + ABG group, whereas the ABG group demonstrated more prominent autologous bone particles and areas of fibrous tissue, Fig. 7.

**Fig. 7:**
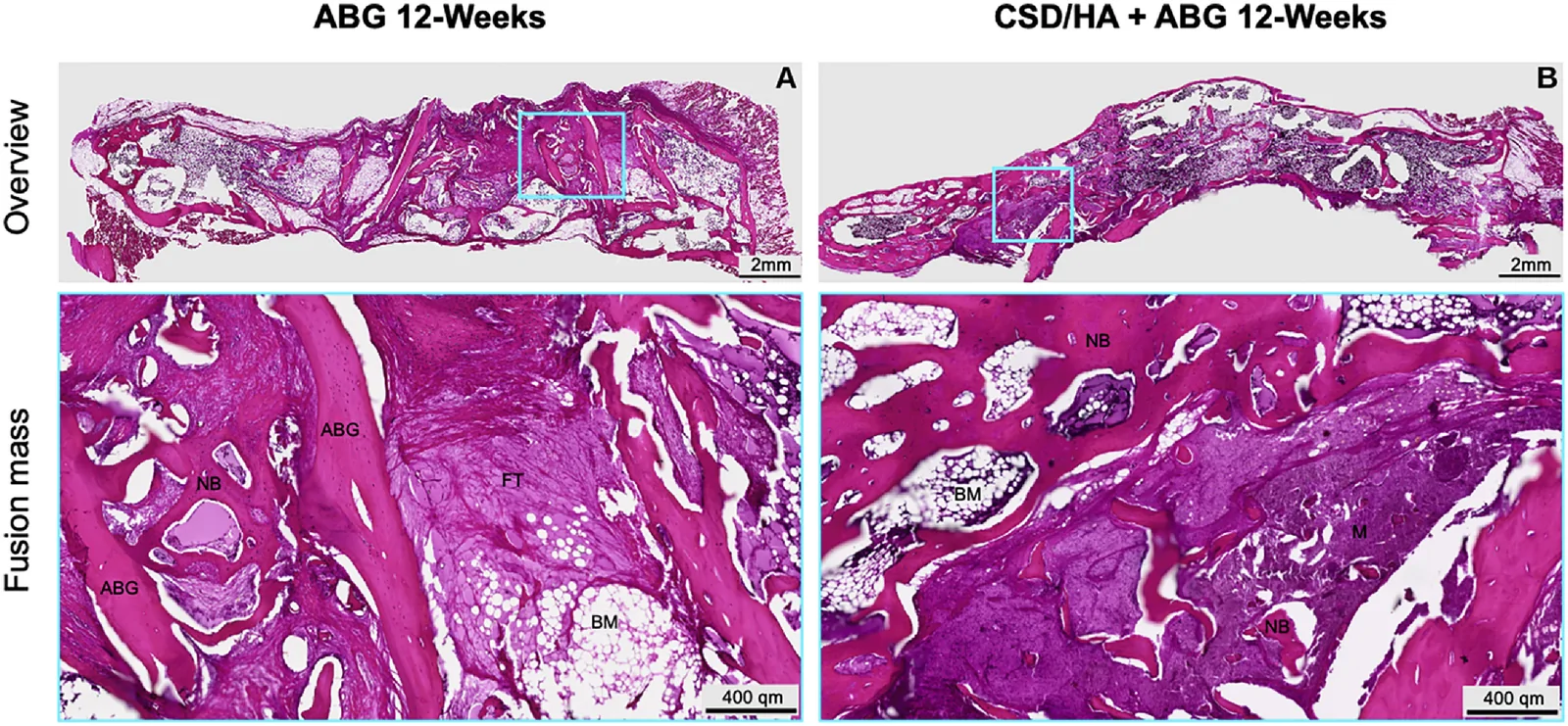
Representative histological comparison of the fusion mass at 12 weeks in the ABG and CSD/HA + ABG groups. (A) Representative ABG specimen showcasing prominent residual allogenic bone particles and intervening fibrous tissue within the fusion mass. (B) Representative CSD/HA + ABG specimen illustrating compact trabecular bone network and areas of new bone partially replacing the biomaterial.

At 12-week time point all samples in the XER + ABG group were classified as fused (100%, 7/7), whereas fusion was observed in 71% (5/7) of samples in the ABG-only group. Representative fused and nonfused specimens shown in, Fig. 8.

**Fig. 8:**
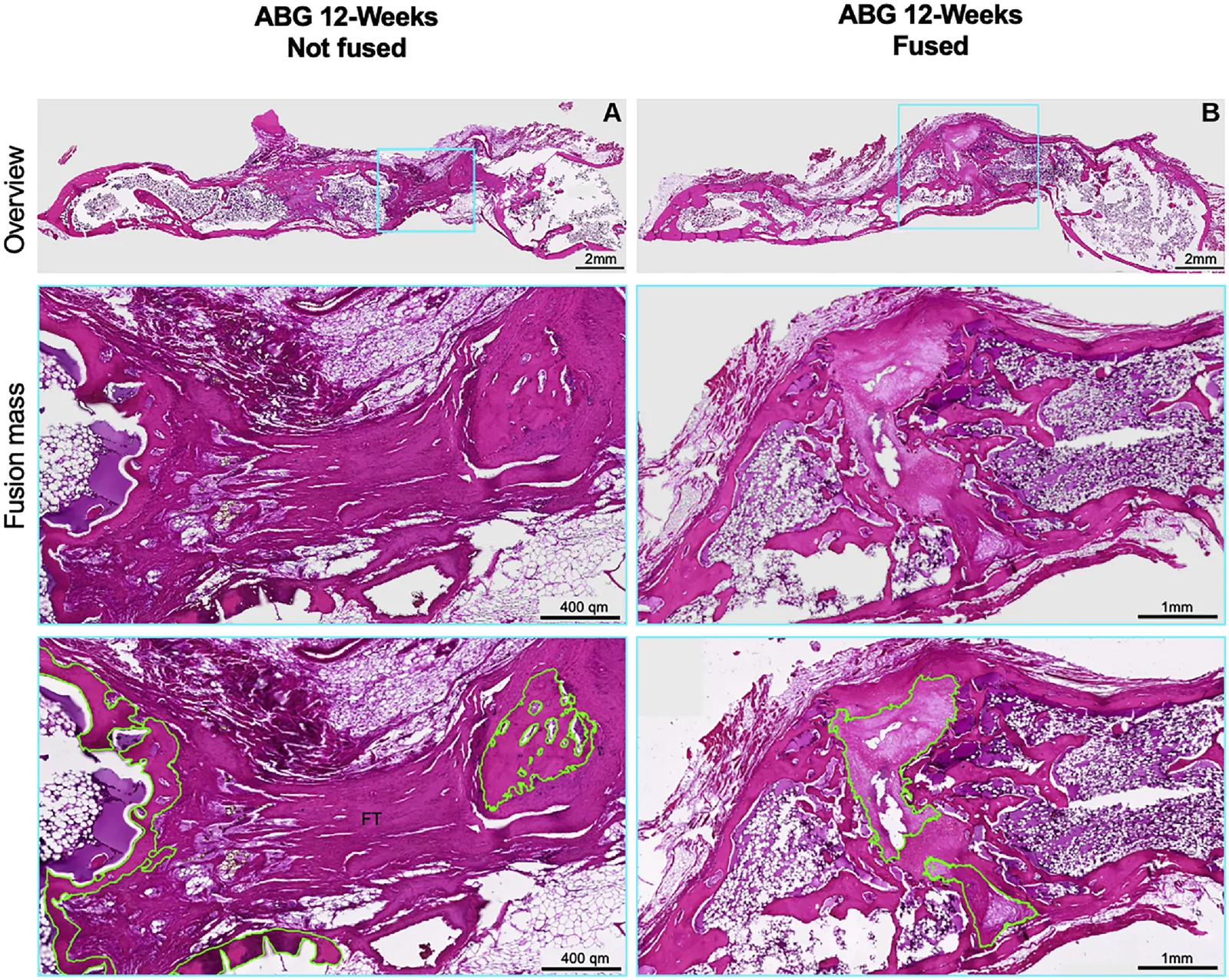
Histological comparison of ABG specimens evaluated as not fused and fused at 12-week endpoint. (A) Representative ABG specimen classified as non-fused, with predominant fibrous tissue (FT) preventing bony bridging (green) in the central region of the fusion mass. (B) Representative ABG specimen classified as fused, showing areas of endochondral ossification (green) that have matured into bone, resulting in a continuous bony bridge across the fusion mass.

### Fusion evaluation

Overall fusion rate was determined by combined results of radiographic, micro-CT, manual palpation data and supported by histological findings, Table 4, Fig. 9. Fusion was classified as positive based on radiographic and micro-CT grading as Lenke grade A (solid bilateral fusion), and absence of motion on manual palpation. Histologically it correlated to continuous trabecular bone or bone marrow seen in the intertransverse region.

**Fig. 9:**
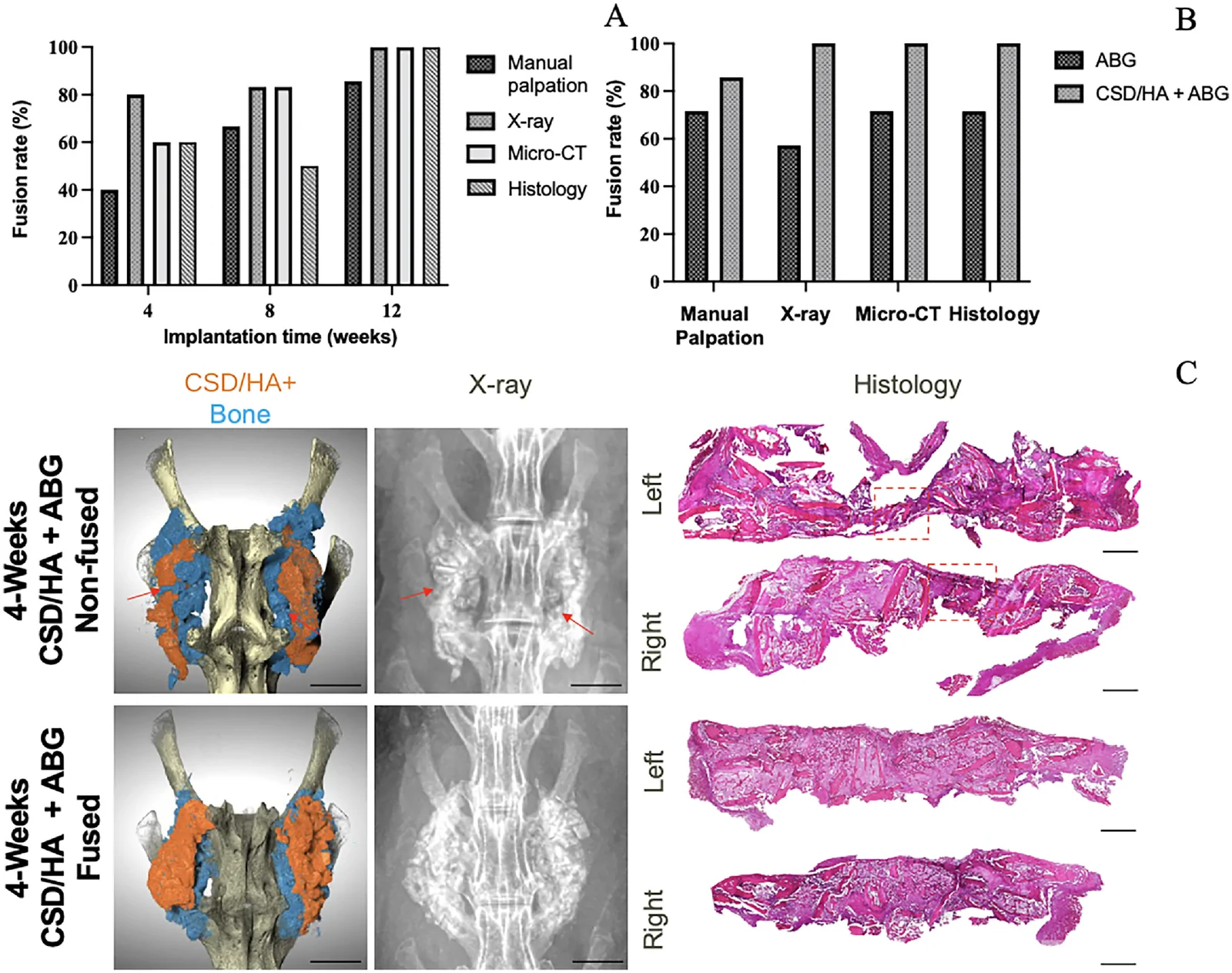
Spinal fusion outcomes assessed using a multimodal evaluation approach, including radiography, micro-CT, manual palpation, and histological assessment. (A) Quantitative analysis of fusion rates in the CSD/HA + ABG group over time, independently evaluated by 3 observers (averaged) using radiographic, manual palpation and histological assessment. (B) Comparison of fusion rates between the CSD/HA + ABG and ABG only groups using 4 fusion assessment methods. (C) Representative specimens classified as non-fused or fused by all 3 fusion assessment methods. Areas of absent bridging are indicated by red arrows or outlines. Scale bars: micro-CT and radiography, 1 cm; histology, 2 mm.

During 4-week implantation time, fusion rate results between mentioned tests varied, reaching 80% fusion rate (4/5) in X-ray, 60% (3/5) in micro-CT and only 40% (2/5) when palpating manually, Fig. 9 (A). Histological evaluation supported fusion in 60% (3/5) of samples at 4 weeks, all of which - except for manual assessment - were consistently classified as fused by the other evaluation methods, Fig. 9 (B). Micro-CT correlated with the radiographic assessment at 8- and 12-week time points, with fusion rate of 83% (5/6) and 100% (7/7), respectively. Although manual palpation data consistently shows lower scores, Fig. 9 (A), histological findings were consistent with radiographic assessment at 12 weeks.

Among samples classified as fused by all evaluation methods, an overall fusion rate of 40% (2/5) was observed in the XER + ABG group at 4 weeks, 33.3% (2/6) at 8 weeks, and 86% (6/7) at 12 weeks, Table 4. Compared to autograft, all fusion evaluation methods (radiographic, micro-CT, manual palpation) combined exhibited higher ratings scores in XER + ABG group at 12 weeks, 86% and 57%, respectively Table 4, Fig. 9 (C). Histological data at 12 weeks indicated complete fusion in the XER + ABG group, consistent with radiographic and micro-CT evaluation (100%, 7/7), whereas in the ABG group histological fusion was consistent with micro-CT and manual palpation findings, with fusion observed in 71% (5/7) of samples. No statistically significant differences were found at any time point.

## Discussion

The main objective of this study was to compare the fusion rate obtained by using XERAFUSE BGE with respect to autologous bone in a well-recognised rabbit PLF model. The secondary objectives were to evaluate the progression of total bone volume and remaining material volume at the experimental time points of 4-, 8-, and 12-weeks. The multimodal analysis of spinal fusion, including planar radiography, micro-CT, manual palpation and histological analysis shows that XERAFUSE BGE produced significantly greater bone volume than autologous bone at 12 weeks, with fusion rates comparable to the autograft control in the tested rabbit PLF model.

Calcium phosphate–based biomaterials chemically resemble apatite in native bone and are available in various forms for bone void filling [15]. In critical bone defects and in orthotopic localization, bone regeneration remains, however, variable and incomplete [11,12]. The controlled resorption exhibited by the studied CSD/HA BGE aligns with the natural bone-healing process. Early release of Ca²⁺ ions has been proposed to create a favourable environment for stem cell recruitment and bone formation, while the remaining HA phase is retained as a scaffold for ongoing bone ingrowth. Previously, CaS-based materials have been shown to dissolve in vivo within 4–8 weeks, a period during which vascular invasion and mineralization play pivotal roles [13]. Microporosity exceeding 100 µm is considered necessary for vascular ingrowth [14], and the porous structure generated during controlled dissolution of the present material is thought to be aligned with these requirements.

The greater bone volume observed with the extender is consistent with the behaviour of the granules over the study period. The pre-set granules occupy and maintain the intertransverse volume, and at 4 weeks the graft and biomaterial held the defect open without central collapse. The calcium sulphate phase then resorbs, biomaterial falling from 41.3±2.0% of the fusion mass at 4 weeks to 28.1±2.6% at 12 weeks, progressively exposing the retained hydroxyapatite framework into which bone grows. New bone was present within the implanted material at 4 weeks, with chondroblastic regions indicating endochondral ossification, and micro-CT showed the granules integrated within a trabecular network by 8 and 12 weeks. The material therefore provides a volume-stable scaffold whose porosity develops in step with bone ingrowth, rather than acting as a passive filler.

However, rapid resorption can adversely affect fusion outcomes. Bone defects subjected to mechanical instability tend to heal through fibrous tissue formation rather than bone regeneration [27]. Support for the importance of phase ratio–dependent resorption kinetics is provided by studies on biphasic calcium phosphate ceramics, in which increasing the proportion of the more soluble β-TCP phase has been associated with enhanced osteoinductive response [28]. Soluble factors, such as Ca²⁺ ions released during dynamic dissolution, may recruit mesenchymal stem cells and stimulate their osteogenic differentiation in a concentration-dependent manner [28]. In the present study, CSD/HA formulation did not exhibit implant-related complications associated with excessive release of calcium or sulphate ions during CSD dissolution [29] and the endochondral bone formation histologically indicates that the stability was sufficient to support bone regeneration via endochondral ossification pathways.

There is a clinical challenge of achieving solid bony union following posterolateral fusion, particularly in geriatric and osteoporotic patients, with nonunion rates up 40% reported [11]. Large animal models incorporating rigid internal fixation have been used to replicate the human surgical scenario [12], with fusion rates exceeding 90% achieved. The rabbit posterolateral fusion model described by Boden provides, however, to our opinion, a more biologically and mechanically stringent environment for assessing the initial in vivo efficacy of graft materials prior to starting clinical evaluation. In this study, the BGE was combined with 1.5 cm³ autograft, a volume considered a credible threshold for testing the effect of test material in comparison with 3 cm³ autograft [25].

The outcomes in the autograft control group are highly consistent with the prior published results using the rabbit PLF model. The 71% (5/7) fusion rate assessed by manual palpation in the control group at 12-weeks in the current study also closely aligns with the pooled autograft fusion rate of 77% (80/104) reported across large series of studies [30]. Most studies report results starting from 6-week endpoint, as fusion success can be 20% lower at earlier time points, but early assessments may detect accelerated healing [10]. In the current study, 40% of fusion rate assessed on manual palpation at 4 weeks, was comparable to 38% reported for autograft controls in large control series [30].

At 8 weeks, 83% of samples in the XER + ABG group were evaluated as fused by both micro-CT and radiographic assessment. Among commercially available calcium phosphate–based bone graft extenders, MASTERGRAFT STRIP has been reported at a comparable level at the 8-week time point, demonstrating a fusion rate of 75% when combined with autograft at a 1:1 ratio; however, the study also reports on adding a significant amount of bone marrow aspirate (Table 1) [15].

At 12 weeks, the current study demonstrates fusion efficacy at least comparable to commercially available bone graft extenders for spinal fusion available at present and previously evaluated under similar preclinical conditions. In the current study, manual palpation at the 12-week endpoint demonstrated a fusion rate of 86% (6/7), which falls within the reported range for ATTRAX Scaffold, MagnetOs Granules, Formagraft, and Catalyst Putty (75–88%) [[11], [12], [13], [14], [15], [30]]. Radiographic and micro-CT evaluation at 12 weeks demonstrated a 100% fusion rate (7/7) in the XER + autograft group, supported by histologic findings. Radiographically, this exceeds fusion rates reported for commercially available bone graft substitutes (63–87.5%) [[11], [12], [13], [14], [15], [30]]. In micro-CT only MagnetOs granules exhibited 100% fusion rates as observed in current study, while others varied 63–75% [[11], [12], [13], [14], [15], [30]].

Importantly, the comparisons depend on the assessment modality used, as spinal fusion outcomes are best interpreted using a multimodal approach, given that no single technique fully captures the fusion process. In the present study, discrepancies were observed between assessment methods: at 4 weeks, fusion rates were 60% by micro-CT and 80% by radiography; at 8 and 12 weeks, micro-CT closely correlated with radiographic evaluation, with fusion rates of 83.3% and 100%, respectively. Manual palpation consistently demonstrated lower fusion rates, with fusion identified in 40%, 67%, and 86% of specimens at 4, 8, and 12 weeks, respectively.

These results can represent the tendency of radiographic superimposition - particularly in the presence of radiopaque materials - to overestimate fusion [19,31]. It is reported that plain radiographs carry an accuracy no greater than 70% for predicting whether a spine fusion is solid [32]. Although manual palpation reflects a clinically relevant assessment of fusion level, it remains inherently subjective and may be influenced by densely packed material rather than true osseous fusion [19]; therefore, correlation with histological findings was important for overall interpretation. Histological evaluation supported fusion in 60% of samples at 4 weeks, 50% at 8 weeks, and 100% at 12 weeks. The closer correlation with manual palpation at 8 weeks and with radiographic findings at 12 weeks underscores the value of a multimodal approach for fusion assessment.

Bone volume, as demonstrated by volumetric micro-CT, increased from 59% at 4 weeks to 63% at 8 weeks and 72% at 12 weeks. At 12 weeks, the bone volume in the BGE group (850±68.4 mm³) increased significantly than in the autograft comparator group (468.9±105.3 mm³). Transient decrease in absolute bone volume observed between 4 and 8 weeks in both groups may reflect early bone remodeling, during which the autograft undergoes resorption and reorganization [27,33].

To our knowledge, this BGE study is the first to use micro-CT data to quantitatively assess 3-dimensional bone volume over time and distinguish bone from residual ceramic material in posterolateral fusion. The 3-dimensional quantitative assessment showed low interobserver variation, providing objective volumetric data, and corroborated with histological evaluations. In instrumented PLF, when using ordinary lower-resolution X-ray or CT for fusion outcome, differentiating new bone from implanted material is difficult, and concordance with histology reported at revision is lacking [34]. Although patient-reported outcome measures including pain indices have shown to positively correlate with radiographic assessment of spinal fusion in general, a critical assessment of this correlation is warranted, particularly when using radio dense materials.

Material degradation was composition-dependent, with 56% resorption observed over 12 weeks, related to the calcium release from CSD. Previous studies have reported rapid dissolution using CaS alone, with degradation occurring within 4–8 weeks; notably, Glazer et al. demonstrated complete resorption within 4 weeks in a rabbit fusion model, resulting in no fusions [5,[35], [36], [37]]. Incorporation of hydroxyapatite in the present CSD/HA BGE likely contributed to a slower and more controlled resorption profile, supporting sustained structural stability during the fusion process.

Several limitations of this study should be acknowledged. Although the preclinical model used demonstrates fusion success rates comparable to those reported in humans, outcomes may differ in other translational animal models or in clinical applications where the bone graft implantation environment, mechanical conditions, and fixation strategies vary. Interpretation of the present findings is therefore subject to limitations related to the choice of model, selected time points, and assessment methods. The BGE was evaluated only at a 1:1 ratio with autograft; alternative ratios or use of the material alone were not investigated and remain outside the scope of this initial assessment. The autograft control was evaluated only at the 12-week endpoint, so the 4- and 8-week data describe the time course of the XER + ABG group alone, and between-group comparison is limited to the terminal timepoint. Inclusion of longer-term endpoints would allow a more comprehensive evaluation of healing progression, fusion maturation, remodeling, and graft resorption. With 5 to 7 specimens per group, fusion rates are therefore reported descriptively, and the similar rates observed in the 2 groups should not be interpreted as statistical equivalence. Fusion was assessed mechanically by manual palpation; quantitative biomechanical testing was not performed, as all specimens were allocated to micro-CT and histological analysis. Residual autograft and newly formed bone share the same mineral phase and cannot be separated by attenuation, so the between-group comparison of bone volume is a comparison of total mineralised bone rather than of new bone formation alone. As the granules resorb, they lose both attenuation contrast and their characteristic geometry, so classification is most reliable at 4 weeks and least reliable at 12 weeks, with residual uncertainty concentrated at resorbing granule margins.

## Conclusion

XERAFUSE a CSD/HA BGE was safe and achieved fusion rates comparable to autograft across all fusion assessment modalities in a 12-week PLF study. The total bone volume in the BGE group was significantly higher compared to ABG only. Collectively, the preclinical findings indicate that a next step would be clinical efficacy studies.

## Author contributions

**G.L.:** investigation, methodology, data curation, formal analysis, writing – original draft, software, visualization. **F.M.:** investigation, methodology, data curation, writing – review and editing, visualization. **J.H.:** investigation, data curation, writing – review and editing. **H.A.:** investigation, writing – review and editing. **D.J.:** investigation. **P.Z.:** investigation. **S.T.:** conceptualization, investigation, supervision, writing – review and editing, funding acquisition. **A.S.:** conceptualization, investigation, supervision, writing – review and editing. **B.D.:** formal analysis, writing – review and editing. **S.Z.:** formal analysis, writing – review and editing. **L.L.:** conceptualization, supervision, funding acquisition, formal analysis, validation, resources, writing – review and editing. **M.T.:** conceptualization, investigation, supervision, funding acquisition, resources, validation, writing – review and editing. **D.B.R.:** conceptualization, investigation, project administration, supervision, funding acquisition, resources, validation, writing – review and editing. All authors have read and agreed to the published version of the manuscript.

## Institutional review board statement

This study was conducted in accordance with national and institutional guidelines and was approved by the relevant Lithuanian Governmental Authority overseeing animal welfare (approval code: G2-277), with the approval granted on 23 April 2024. The study followed the Animal Research: Reporting of In Vivo Experiments (ARRIVE) guidelines to ensure transparent and ethical reporting of all in vivo procedures.

## Data availability statement

The original contributions presented in this study are included in the article. Further inquiries can be directed to the corresponding author.

## Declaration of competing interest

DBR, MT and LL are co-founders and hold stocks in Moroxite AB, a non-listed company based in Lund, Sweden.

## Appendix Supplementary materials

Image, image 1 dummy alt text

Image, image 2 dummy alt text

Image, image 3 dummy alt text

Image, image 4 dummy alt text

Image, image 5 dummy alt text

Image, image 6 dummy alt text

Image, image 7 dummy alt text

Image, image 8 dummy alt text

Image, image 9 dummy alt text

Image, image 10 dummy alt text

Image, image 11 dummy alt text

Image, image 12 dummy alt text

Image, application 13
