# Supplementary material for: Evaluation of a pre-set calcium sulphate dihydrate/hydroxyapatite bone graft extender in a rabbit posterolateral spinal fusion model
Source: N Am Spine Soc J. 2026 Oct 6;28:100955. doi: 10.1016/j.xnsj.2026.100955 (PMC13755484; doi:10.1016/j.xnsj.2026.100955)
Supplement: Supplementary file 1 [file mmc13.docx]

**Figure S1_1** CSD/HA+ABG. Comparison of X-ray fusion, 4 vs. 8 vs. 12 weeks.

**Figure S1_2** CSD/HA+ABG vs. ABG. Comparison of Bone Volume, 12-weeks.

**Figure S2_1** CSD/HA+ABG, 4-Weeks.

Note:

CSD/HA only = Orange color represents only material after removing autologous bone from the material.

Bone only = Blue color represents a mix of autologous bone/new bone only. The material has been removed. NOT TO BE CONFUSED WITH THE 100% autologous bone group. CSD/HA+Bone = Combination of CSD/HA+Bone (autograft+new bone) as an overlap.

Scale bar: 1 cm (applies to all histological images).

**Figure S2_2** CSD/HA+ABG, 8-Weeks.

**Figure S2_3** CSD/HA+ABG, 12-Weeks.

**Figure S2_4** CSD/HA+ABG. Comparison of remaining material, 4 weeks vs. 8 weeks vs. 12 weeks.

**Figure S2_5**

Change in material volume over time. Left shows the absolute material volume in mm3 and right shows % of remaining material. * indicates p<0.05, *** indicates p<0.001 and **** p<0.0001.

Change in bone volume over time. Left shows the absolute bone volume in mm3 and right shows % of remaining bone. * indicates p<0.05 and **** p<0.0001.

**Figure S2_6** CSD/HA+ABG vs. ABG. Comparison of Bone Volume, 12-weeks.

**Figure S3_1** CSD/HA+ABG, 4-Weeks. Scale bar: 2 mm

**Figure S3_2** CSD/HA+ABG, 8-Weeks. Scale bar: 2 mm

**Figure S3_3** CSD/HA+ABG, 12-Weeks. Scale bar: 2 mm

**Figure S3_4** ABG, 12-Weeks. Scale bar: 2 mm
